# Serum creatinine to cystatin C ratio and clinical outcomes in adults with non-dialysis chronic kidney disease

**DOI:** 10.3389/fnut.2022.996674

**Published:** 2022-09-26

**Authors:** Young Youl Hyun, Kyu-Beck Lee, Hyoungnae Kim, Yaeni Kim, Wookyung Chung, Hayne Cho Park, Seung Hyeok Han, Yun Kyu Oh, Sue Kyung Park, Kook-Hwan Oh

**Affiliations:** ^1^Division of Nephrology, Department of Internal Medicine, Kangbuk Samsung Hospital, Sungkyunkwan University School of Medicine, Seoul, South Korea; ^2^Division of Nephrology, Seoul Hospital, Soonchunhyang University, Seoul, South Korea; ^3^Department of Internal Medicine, Seoul St. Mary’s Hospital, The Catholic University, Seoul, South Korea; ^4^Department of Internal Medicine, Gil Hospital, Gachon University, Incheon, South Korea; ^5^Department of Internal Medicine, Kangnam Sacred Heart Hospital, Hallym University Medical Center, Seoul, South Korea; ^6^Department of Internal Medicine, College of Medicine, Yonsei University, Seoul, South Korea; ^7^Department of Internal Medicine, Boramae Hospital, Seoul National University, Seoul, South Korea; ^8^Department of Preventive Medicine, Seoul National University College of Medicine, Seoul, South Korea; ^9^Department of Internal Medicine, Seoul National University College of Medicine, Seoul National University Hospital, Seoul, South Korea

**Keywords:** creatinine/cystatin C ratio, death, cardiovascular events, chronic kidney disease, muscle wasting

## Abstract

**Background:**

Studies have suggested that the serum creatinine/cystatin C (Cr/CysC) ratio is a surrogate marker for muscle wasting is associated with adverse outcomes in several disease conditions. To clarify the utility of the Cr/CysC ratio as a prognostic marker in chronic kidney disease (CKD) we evaluated the association between the Cr/CysC ratio clinical outcomes in patients with non-dialysis CKD.

**Methods:**

This prospective observational cohort study included 1,966 participants of the KoreaN cohort study Outcomes in patients With CKD (KNOW-CKD). We evaluated associated factors with the serum Cr/CysC ratio and association between the serum Cr/CysC ratio and composite outcomes of all-cause death and cardiovascular events (CVEs).

**Results:**

The mean age was 54 ± 12 (SD) years and 61% were men. The mean serum Cr/CysC ratio was 10.97 ± 1.94 in men and 9.10 ± 1.77 in women. The Cr/CysC ratio correlated positively with urinary creatinine excretion, a marker of muscle mass. In the fully adjusted Cox proportional hazard model, the Cr/CysC ratio was associated with the occurrence of adverse outcomes through a median follow-up of 5.9 years [hazard ratio (HR) = 0.92, 95% confidence interval (CI) = 0.85–0.99 for the composite outcomes, HR = 0.87, 95% CI, 0.78 − 0.97 for all-cause death, and HR = 0.93; 95% CI, 0.84–1.04 for CVEs]. In subgroup analyses, there were interactions of the Cr/CysC ratio with age and sex for risk of the clinical outcomes, but not eGFR group.

**Conclusion:**

A higher Cr/CysC ratio is associated with a lower risk of the composite outcomes, especially all-cause mortality, even after adjusting for eGFR. These suggest that the Cr/CysC ratio is a useful prognostic marker in CKD.

## Introduction

Chronic kidney disease (CKD) is a prevalent condition worldwide that contributes an important risk of morbidity and mortality (1). CKD is also an important risk factor for protein-energy wasting (2). Uremia in CKD leads to accelerated muscle protein breakdown combined with low dietary energy and protein intakes (3, 4). Muscle wasting induces mobility limitations, loss of independence, and vulnerability to disease complications. Muscle wasting is an important determinant of adverse outcomes in CKD. Several methods are available to measure muscle mass, including imaging techniques, anthropometric parameters, and biochemical markers. Imaging studies such as dual-energy X-ray absorptiometry (DXA) are a standard method for assessing muscle mass (5). In adults with CKD, DXA is a standard method to measure body composition despite being influenced by volume status (6). However, a wide range of methods can be used to assess muscle mass. Availability, cost, and ease of use can determine whether techniques are better suited to clinical practice or research.

Serum creatinine and cystatin C are well-established markers of kidney function (7). Creatinine is generated in proportion to muscle mass, but cystatin C is not affected by muscle mass (8). Loss of muscle mass during the wasting process of CKD is accompanied by a decline in serum creatinine, but not in cystatin C. We postulated the serum Cr/CysC ratio is a surrogate marker for muscle wasting in CKD. Previous studies have reported that the Cr/CysC ratio was associated with muscle mass in patients who were critically ill (9), older adults (10), or had several chronic diseases. Moreover, the Cr/CysC ratio was associated with clinical outcomes in patients in the intensive care unit (9) and those receiving continuous kidney replace therapy (11). A cross-sectional study of patients with non-dialysis CKD reported that Cr/CysC was independently associated with skeletal muscle mass and strength (12). They suggested that Cr/CysC could be a surrogate marker for detecting muscle wasting in CKD. However, the implication of Cr/CysC in the clinical outcomes of CKD are uncertain.

To clarify the utility of the serum Cr/CysC ratio as a prognostic markers in CKD, we analyzed a prospective cohort dataset from the KoreaN cohort study for Outcome in patients With CKD (KNOW-CKD). In this study, we evaluated factors associated with the serum Cr/CysC ratio and the association between the serum Cr/CysC ratio and composite outcomes of all-cause death and cardiovascular events (CVEs) in adults with non-dialysis CKD.

## Materials and methods

### Study participants and design

The KNOW-CKD (ClinicalTrials.gov identifier NCT01630486) is a nationwide prospective cohort study investigating the clinical outcomes of Koreans with non-dialysis dependent CKD (13). Between 2011 and 2016, a total of 2,238 adults age 20–75 years with non-dialysis CKD stage G1-G6 were enrolled from nine tertiary care hospital. Subjects were excluded if they had a history of malignancy, advanced heart failure, a single kidney, liver cirrhosis, chronic lung disease or other factors according to the study protocol. We analyzed 1,966 participants from this cohort who underwent extensive laboratory tests, completed a health questionnaire, and for whom follow-up data were available (Supplementary Figure 1). This study protocol was approved by the Institutional Review Board of the participating centers. Informed consents were obtained from all participants.

### Data collection and measurements

Baseline demographic characteristics, medical history, and lifestyle factors were collected by self-report and a review of the medical records. A history of hypertension was defined as a self-reported history of hypertension or current use of antihypertension medication. Diabetes mellitus was defined as a fasting serum glucose level ≥ 126 mg/dL, a history of diabetes, or current use of antidiabetic medication. Urinary albumin excretion was determined using the spot urine albumin-to-creatinine ratio (ACR).

### Main exposure of interest

Sample for serum creatinine and cystatin C were collected at baseline after overnight fasting. Serum creatinine was measured using an isotope dilution mass spectrometry-calibrated method and cystatin C was measured using immunonephelometry with calibration against a reference at a central lab. The estimated glomerular filtration rate (eGFR) was calculated by using the CKD Epidemiology Collaboration (CKD-EPI) creatinine equation (14). The serum Cr/CysC ratio (creatinine in mg/L to cystatin C in mg/L) was calculated from the values measured concomitantly at baseline. Because the serum Cr/CysC ratio was differed by sex, we categorized the participants into male and female quartiles according to Cr/CysC ratio.

### Study outcomes

Patients were followed up until their last visit, initiation of renal replacement therapy, or death before March 31, 2021. All-cause death or CVE during follow up was the primary outcomes. CVE was defined as the occurrence of a fatal or non-fatal CVE during follow up including any coronary artery events (unstable angina, myocardial infarction, coronary intervention, or coronary surgery), hospitalization for heart failure, ischemic or hemorrhagic stroke, or symptomatic arrhythmia. The composite outcome of all-cause death and CVEs was assessed.

### Statistical analysis

**FIGURE 1:**
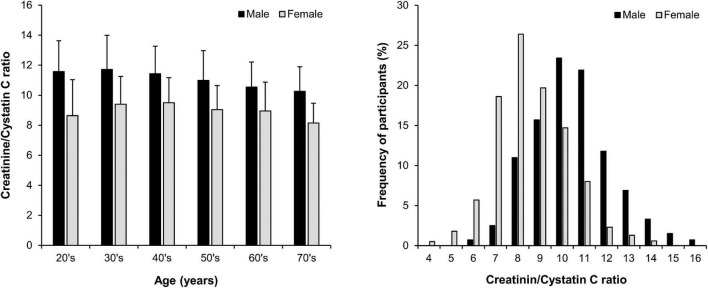
Distribution of the serum creatinine/cystatin C ratio (mean ± SD) by age and sex in 1,966 participants with non-dialysis chronic kidney disease.

We initially considered our primary predictor variable, Cr/CysC ratio, as a continuous variable. The Cr/CysC ratio in males was greater than that in females (Figure 1). We thus divided the study population into sex-specific quartiles for descriptive purposes in the analyses. We described the baseline characteristics across group using the mean ± standard deviation or median and interquartile range for continuous variables and number with percent for categorical variables.

For cross-sectional analyses at baseline, we used a linear regression model of the Cr/CysC ratio controlled for demographic, comorbid, and laboratory factors. We initially considered unadjusted models and then adjusted for age, sex, eGFR, and ACR. In the full adjusted model, we also adjusted for comorbidity and laboratory data.

Cumulative event probabilities were estimated using a Kaplan-Meier analysis and log-rank tests. Cox proportional hazard models were developed to determine the association between Cr/CysC ratio and composite outcomes, all-cause death, and CVEs. The Cr/CysC ratio was evaluated as a continuous variable and a categorical variable of quartiles. The data were expressed as hazard ratio (HR) with 95% confidence interval (CI). Model 1 considered baseline age, sex, eGFR, and the natural log of ACR. Model 2 added systolic blood pressure, body mass index, C-reactive protein, serum albumin, and a history of diabetes or cardiovascular disease. Moreover, to assess their effects on our findings, we tested for associations between the Cr/CysC ratio and outcomes stratified by age, sex, and eGFR group. The Cox model for all-cause death and CVEs was used in cubic spline analyses, with each curve having four equally distributed knots, placed at the 5th, 35th, 65th, 95th percentiles of the Cr/CysC ratio. The cubic spline model used a Cr/CysC ratio of 7.0 as reference value. All analyses were performed using Stata version 17 (STATA Corp.).

## Results

### Baseline characteristics and patient outcomes

The baseline characteristics of the study participants are presented according to the sex-specific quartiles of Cr/CysC ratio (Table 1). The average age was 54 ± 12 years, 1,190 males (61%), and eGFR was 54 ± 12 mL/min/1.73 m^2^. The distribution of Cr/CysC ratio by age and sex is show in Figure 1. The average serum Cr/CysC ratio of males was 10.97 ± 1.94, and that of females was 9.10 ± 1.77. Compared with participants in quartile 1, those in the higher quartiles were younger, and more likely to be non-smokers, and have no history of diabetes or cardiovascular diseases. They were also likely to have higher 24-h urine creatinine and serum albumin values.

**TABLE 1:** Baseline characteristics according to serum Cr/CysC ratio quartiles in 1,966 adults with chronic kidney disease.

		Quartile of creatinine/Cystatin C ratio				
Variables	Total (*n* = 1,966)	Q1 (*n* = 492)	Q2 (*n* = 491)	Q3 (*n* = 492)	Q4 (*n* = 491)	*P* for trend
Male range	6.45–24.14	6.45–9.69	9.69–10.81	10.81–11.97	11.98–24.14	
Female range	4.62–23.49	4.62–7.90	7.90–8.91	8.91–10.11	10.14–23.49	
Age, years	53.6 ± 12.3	56.7 ± 12.4	55.7 ± 11.4	52.5 ± 11.9	49.4 ± 12.06	< 0.001
Male sex	1,190 (61%)	298 (61%)	297 (61%)	298 (61%)	297 (61%)	0.9
BMI, kg/m^2^	24.57 ± 3.89	24.70 ± 3.82	24.60 ± 3.23	24.57 ± 3.20	24.43 ± 3.27	0.2
Creatinine, mg/dL	1.81 ± 1.15	1.44 ± 0.69	1.63 ± 0.83	1.78 ± 0.91	2.37 ± 1.69	< 0.001
Cystatin C, mg/L	1.75 ± 0.92	1.77 ± 0.80	1.70 ± 0.83	1.68 ± 0.85	1.85 ± 1.14	0.2
eGFR, mL/min/1.73m^2^	53.6 ± 31.0	61.0 ± 30.8	54.8 ± 29.7	52.5 ± 30.4	46.0 ± 31.4	< 0.001
UACR, mg/g	348 [78–1053]	462 [131–1444]	306 [64–1049]	312 [71–940]	331 [66–972]	0.07
24-h U creatinine, mg/day	1,177 ± 412	1,087 ± 383	1,154 ± 374	1,188 ± 409	1,273 ± 455	< 0.001
Systolic BP, mmHg	128 ± 16	129 ± 17	128 ± 14	127 ± 16	128 ± 17	0.07
Diastolic BP, mmHg	77 ± 11	77 ± 12	77 ± 11	77 ± 11	77 ± 11	0.2
C-reactive protein, mg/L	0.6 [0.2–1.7]	0.8 [0.3–2.2]	0.7 [0.3–1.8]	0.6 [0.2–1.5]	0.5 [0.2–1.3]	<0.001
Albumin, g/dL	4.19 ± 0.42	4.08 ± 0.48	4.23 ± 0.40	4.21 ± 0.38	4.23 ± 0.41	<0.001
Hemoglobin, g/dL	12.8 ± 2.0	12.7 ± 1.9	13.0 ± 1.9	13.0 ± 1.9	12.7 ± 2.2	0.7
Diabetes	665 (34%)	191 (39%)	181 (37%)	148 (30%)	145 (30%)	<0.001
Hypertension	1,889 (96%)	478 (97%)	471 (96%)	474 (96%)	499 (95%)	0.3
Cardiovascular disease	309 (16%)	91 (19%)	102 (21%)	69 (14%)	47 (10%)	<0.001
Current smoker	294 (15%)	99 (20%)	68 (14%)	77 (16%)	50 (10%)	<0.001

Continuous variables expressed as mean ± standard deviation or median [interquartile range]; categorical variables, as number (percentage). BMI, body mass index; BP, blood pressure; eGFR, estimated glomerular filtration rate; UACR, urine albumin-creatinine ratio.

A total of 258 composite outcomes occurred: 130 all-cause of deaths and 163 CVEs occurred during a median follow-up of 5.9 years. The incidence rates of composite outcomes were 34.2, 24.5, 21.1, and 14.4/1,000 person-years according to quartiles, respectively. The trends in the rate of the composite outcomes, all-cause death, and CVEs were all statistically significant (*P* < 0.01) (Table 2).

**TABLE 2:** Incidence of the composite outcomes, all-cause death, and cardiovascular events according to quartile of CysC/Cr ratio.

		Quartile of creatinine/Cystatin C ratio				
Outcomes	Total (*n* = 1,966)	Q1 (*n* = 492)	Q2 (*n* = 491)	Q3 (*n* = 492)	Q4 (*n* = 491)	*P* for trend
No. of person-years	11,033	2,601	2,781	2,887	2,764	
**Composite outcomes**						
No of incidence	258	89	68	61	40	
Incidence rate (1,000 person-year)	23.4	34.2	24.5	21.1	14.4	<0.001
**All-cause death**						
No. of incidence	130	46	33	31	20	
Incidence rate (1,000 person-year)	11.8	17.7	11.9	10.7	7.2	<0.001
**Cardiovascular events**						
No. of incidence	163	55	43	40	25	
Incidence rate (1,000 person-year)	14.8	21.1	15.5	13.9	9.0	0.01

### The serum creatinine/cystatin C ratio and clinical parameters at baseline

The Cr/CysC ratio had a week negative correlation with the natural log of C-reactive protein and a weak positive correlation with serum albumin. The Cr/CysC ratio had a significant positive correlation with 24-h urine creatinine (*r* = 0.376, *P* < 0.001) (Supplementary Figure 2).

In multivariable linear regression analysis, the Cr/CysC ratio had a negative association with age, female sex, eGFR, cardiovascular disease, current smoking, and the natural logs of ACR and CRP. Cr/CysC ratio had a positive association with serum albumin and 24-h urine creatinine (coefficient of determinant, *R*^2^ = 0.391) (Supplementary Table 1).

### The serum creatinine/cystatin C ratio and the composite outcomes, all-cause death, and cardiovascular events

The Kaplan-Meier curves revealed that the cumulative probabilities of the composite outcomes, all-cause death, and CVEs were significantly lower among patients in quartile 1 (Q1) of baseline Cr/CysC ratio compared with other quartiles (log-rank *P* < 0.01) (Supplementary Figure 3).

The association of Cr/CysC ratio with composite outcomes, all-cause death, and CVEs were evaluated using multivariable Cox proportional hazards regression analyses (Table 3). The HRs of the Cr/CysC ratio as a continuous variable for composite outcome, all-cause death, and CVEs were 0.92 (95% CI, 0.85 − 0.99, *P* = 0.05), 0.87 (95% CI, 0.78 − 0.97, *P* = 0.02), and 0.93 (95% CI, 0.84 − 1.04, *P* = 0.2), respectively, in model 2. The HRs for Q4, the quartile with the highest Cr/CysC ratio, for the composite outcome, all-cause death, and CVEs were 0.69 (95% CI, 0.45 − 0.99, *P* = 0.05), 0.54 (95% CI, 0.30 − 0.97, *P* = 0.04), and 0.72 (95% CI, 0.43 − 1.21, *P* = 0.2), respectively, in model 2.

**TABLE 3:** Hazard ratio for death and cardiovascular events based on the Cr/CysC ratio in 1,966 adults with chronic kidney disease.

	Crude		Model 1		Model 2	
	Hazard ratio (95% CI)	*P*	Hazard ratio (95% CI)	*P*	Hazard ratio (95% CI)	*P*
**Composite outcomes**						
Continuous (per 1 unit Cr/CysC)	0.93 (0.87–0.99)	0.02	0.87 (0.80–0.95)	0.01	0.92 (0.85–0.99)	0.05
**Categorical**						
Q1	1.00 (reference)		1.00 (reference)		1.00 (reference)	
Q2	0.67 (0.48–0.92)	0.01	0.75 (0.53–1.01)	0.08	0.82 (0.59–1.14)	0.2
Q3	0.53 (0.38–0.74)	<0.001	0.62 (0.44–0.87)	0.01	0.71 (0.50–1.01)	0.06
Q4	0.39 (0.27–0.53)	<0.001	0.55 (0.37–0.82)	0.01	0.69 (0.45–0.99)	0.05
**All-cause death**						
Continuous (per 1 unit Cr/CysC)	0.94 (0.86–1.02)	0.1	0.83 (0.74–0.93)	0.01	0.87 (0.78–0.97)	0.02
**Categorical**						
Q1	1.00 (reference)		1.00 (reference)		1.00 (reference)	
Q2	0.68 (0.42–1.02)	0.06	0.66 (0.42–1.04)	0.08	0.78 (0.49–1.23)	0.3
Q3	0.58 (0.37–0.91)	0.02	0.57 (0.35–0.91)	0.02	0.68 (0.42–1.10)	0.1
Q4	0.39 (0.23–0.66)	<0.001	0.42 (0.24–0.74)	0.01	0.54 (0.30–0.97)	0.04
**Cardiovascular events**						
Continuous (per 1 unit Cr/CysC)	0.92 (0.85–1.00)	0.05	0.89 (0.80–0.99)	0.04	0.93 (0.84–1.04)	0.2
**Categorical**						
Q1	1.00 (reference)		1.00 (reference)		1.00 (reference)	
Q2	0.67 (0.45–0.99)	0.05	0.79 (0.52–1.19)	0.3	0.80 (0.53–1.21)	0.3
Q3	0.53 (0.35–0.81)	0.01	0.67 (0.43–1.03)	0.07	0.72 (0.46–1.12)	0.1
Q4	0.38 (0.24–0.61)	<0.001	0.60 (0.36–1.00)	0.05	0.72 (0.43–1.21)	0.2

Model 1, adjusted for age, sex, estimated glomerular filtration rate, and natural log of albuminuria. Model 2, additionally adjusted for diabetes, cardiovascular disease, body mass index, systolic blood pressure, current smoking, albumin, and the natural log of C-reactive protein. CI, confidence interval.

**FIGURE 2:**
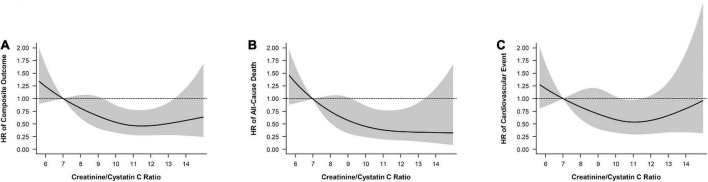
Cubic spline model shows relationship of creatinine/cystatin C ratio with **(A)** composite outcomes, **(B)** all-cause death, and **(C)** cardiovascular events. Adjustments were made for model 2 variables (age, sex, eGFR, ACR, diabetes, cardiovascular disease, body mass index, smoking, albumin, C-reactive protein).

The associations between the Cr/CysC ratio and the composite outcome, all-cause death, and CVEs were showed using a cubic spline analysis. The risks for the composite outcome, all-cause death, and CVEs were lower with greater Cr/CysC ratio. The risk for all-cause death became progressively lower as the Cr/CysC ratio increased (Figure 2).

### Subgroup analysis

**FIGURE 3:**
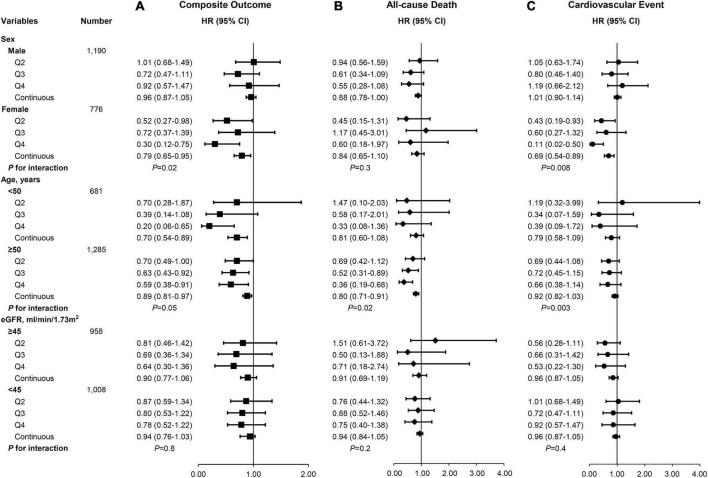
There were different risks of **(A)** composite outcomes, **(B)** all-cause death, and **(C)** cardiovascular events according to subgroups in the multivariable-adjusted Cox proportional hazards model (Model 2).

We further examined the effect of modification of the Cr/CysC ratio on risk of the composite outcome, all-cause death, and CVEs in several subgroups (Figure 3). The association between the Cr/CysC ratio and the composite outcome, all-cause death, and CVEs was consistent across eGFR subgroups (< 45 vs. ≥ 45 mL/min/1.73 m^2^). The relationship between the Cr/CysC ratio quartile and composite outcomes was attenuated in younger adults (age < 50 years) (*P* for interaction = 0.05). The relationship between the Cr/CysC ratio quartile and composite outcomes was attenuated in males (*P* for interaction = 0.02). We found significant interactions among subgroups by age and sex.

## Discussion

In this study, we found that the serum Cr/CysC ratio was associated with the risk of a composite outcome of all-cause death and CVEs in adults with non-dialysis CKD, regardless of kidney function. The serum Cr/CysC ratio had a significantly positive correlation with urinary creatinine excretion, a muscle mass marker (15). A higher Cr/CysC ratio was strongly associated with a lower risk of all-cause death and that association was independent of demographics, comorbidities, and clinical factors at baseline. Among the subgroups of patients, the association between the Cr/CysC ratio and the composite outcomes was consistent across eGFR subgroups. These findings suggest that the Cr/CysC ratio could be a prognostic marker of clinical outcomes in CKD.

Serum creatinine and cystatin C are widely used endogenous glomerular filtration markers (7). Creatinine is an end product of muscle catabolism. Its main non-GFR determinants include muscle mass and protein intake, and it varies substantially by age, sex, and chronic illness (16, 17). Cystatin C is low-molecular weight protein enzymes produced by nucleated cells that is involved in the inflammatory cascade. Its serum concentration can be affected by non-GFR determinants such as inflammation, cardiovascular disease, obesity, and smoking (18, 19).

Previous studies reported a positive correlation between the Cr/CysC ratio and muscle mass in patients with intensive care unit (9), older adults (10), type 2 diabetes (20), chronic lung disease (21), or cancer (22). Kashani et al. (9) defined the Cr/CysC ratio as a sarcopenic index that correlated significantly with muscle mass measured abdominal CT scan. As a sarcopenic index, it predicted mortality in 226 patients receiving intensive care. Moreover, a recent study reported that the Cr/CysC ratio correlated with muscle quality (myosteatosis) and physical performance in older adults, independent of muscle mass (23). The Cr/CysC ratio was associated with major adverse CVEs in patients with obstructive coronary artery disease. In patients receiving intensive care and continuous kidney replacement therapy, a higher Cr/CysC ratio was associated with longer survival (11). In a cross-sectional study of 272 patients with CKD, the Cr/CysC ratio correlated with skeletal muscle mass and hand grip strength, and appeared to be a surrogate marker for muscle wasting (12). In our study, serum Cr/CysC was independently and positively correlated with urine creatinine excretion. Thus, Cr/CysC could be represent as a muscle wasting marker in CKD.

For reasons similar to those put forward for creatinine and cystatin C, a larger difference between cystatin C- and creatinine-eGFR has been associated with lower frailty, injurious falls, hospitalization, CVEs, and mortality in adults with hypertension of a cohort of the Systolic Blood Pressure Intervention Trial (SPLINT) (24). Kim et al. (25) reported that a positive difference between cystatin C- and creatinine-eGFR in the KNOW-CKD cohort was associated with a higher risk of CVEs and accelerated coronary artery calcification. However, information about the association between the straight-forward serum Cr/Cys C ratio and long-term clinical outcomes from CKD is limited. In this study, we showed that a higher Cr/CysC ratio was associated with a lower risk of CVEs and all-cause death. Among the subgroups of patients, we found significant interaction among subgroups by age and sex, but no interaction among subgroups by eGFR.

Patients with CKD have many risk factor for muscle wasting, including poor appetite, inflammation, insulin resistance, and metabolic acidosis (2, 3). Muscle wasting is thus relevant in CKD, but it goes underdiagnosed. There are several methods for assessing muscle mass with imaging, such as DXA, computed tomography or magnetic resonance imaging (4, 5). However, those tests are costly, entail radiation, and are not available in all clinical settings. Bioimpedance is an inexpensive alternative for assessing body composition, but it is greatly influenced by hydration status and limb size in CKD. Instead, we suggest the serum Cr/Cys C ratio, which is readily available and time effective for capturing sarcopenia and serve as a biomarker of adverse outcomes in CKD.

This study has several limitations. First, in the KNOW-CKD cohort, all-cause death, CVEs, and the composite outcomes occurred in only 130 (6.6%), 163 (8.3%), and 258 (13.1%) patients, respectively, which is lower than in other CKD cohorts. We previously showed that our cohort had a lower cardiovascular risk burden than other cohorts (26, 27) and our lower event rate could have decreased the statistical power of our results. Second, we adjusted for several clinical factors in multivariable analyses, but other factors might also influence serum creatinine and cystatin C levels. For example, we did not evaluate protein intake, volume status, exercise habits, medications, and thyroid function. Third, we did not have data about sarcopenia. We did not measure muscle mass using an image analysis or muscle function by grip strength or walking speed. We therefore could not directly investigate the association between the serum Cr/Cys C and sarcopenia in CKD. Forth, our study participants were all Korean patients with CKD. Sex and age modified the association between Cr/CysC ratio and clinical outcomes. A recent study reported that a higher Cr/CysC ratio was associated with lower mortality in both non-black and black race people. However, the effect was more significant among black people (28). Therefore, it might be difficult to generalize our findings to all patients with CKD. Further studies are required to extrapolate our present findings. Despite those limitations, our study has several strengths. We used comprehensive health history and laboratory data from the nationwide KNOW-CKD cohort. All blood samples were sent to a single central laboratory for accurate measurement of serum creatinine and cystatin C. We found that the serum Cr/Cys C ratio is a simple marker for clinical outcomes. The serum Cr/CysC level was associated with 24-h urine creatinine, albumin and CRP, and might be link for muscle mass, nutrition, and inflammation in CKD.

## Conclusion

In conclusion, the serum Cr/Cys C ratio is associated with the risk of all-cause of death and CVEs among adults with non-dialysis CKD. These findings suggest that the Cr/CysC ratio could be used a prognosis marker for adults with non-dialysis CKD. Further evaluations are needed for its generalized application of our results.

## Data availability statement

Publicly available datasets were analyzed in this study. This data can be found here: KNOW-CKD data. And I did not detect any particular expressions.

## Ethics statement

The studies involving human participants were reviewed and approved by Kangbuck Samsung Hospital IRB. The patients/participants provided their written informed consent to participate in this study.

## Author contributions

YH, K-BL, and YK: research idea and study design. WC, SH, and K-HO: data acquisition. HK and SP: statistical analysis. K-BL, HP, YK, and K-HO: supervision and mentorship. All authors have read and approved the final version of the manuscript.

## References

[1] Gbd Chronic Kidney Disease Collaboration. Global, regional, and national burden of chronic kidney disease, 1990-2017: a systematic analysis for the Global burden of disease study 2017. *Lancet.* (2020) 395:709–33. 10.1016/s0140-6736(20)30045-3 32061315 PMC7049905

[2] Hanna RM Ghobry L Wassef O Rhee CM Kalantar-Zadeh K. A practical approach to nutrition, protein-energy wasting, sarcopenia, and cachexia in patients with chronic kidney disease. *Blood Purif.* (2020) 49:202–11. 10.1159/000504240 31851983

[3] Stenvinkel P Carrero JJ von Walden F Ikizler TA Nader GA. Muscle wasting in end-stage renal disease promulgates premature death: established, emerging and potential novel treatment strategies. *Nephrol Dial Transplant.* (2016) 31:1070–7. 10.1093/ndt/gfv122 25910496

[4] Carrero JJ Johansen KL Lindholm B Stenvinkel P Cuppari L Avesani CM. Screening for muscle wasting and dysfunction in patients with chronic kidney disease. *Kidney Int.* (2016) 90:53–66. 10.1016/j.kint.2016.02.025 27157695

[5] Buckinx F Landi F Cesari M Fielding RA Visser M Engelke K Pitfalls in the measurement of muscle mass: a need for a reference standard. *J Cachexia Sarcopenia Muscle.* (2018) 9:269–78. 10.1002/jcsm.12268 29349935 PMC5879987

[6] Ikizler TA Burrowes JD Byham-Gray LD Campbell KL Carrero JJ Chan W KDOQI clinical practice guideline for nutrition in CKD: 2020 update. *Am J Kidney Dis.* (2020) 76:S1–107. 10.1053/j.ajkd.2020.05.006 32829751

[7] Rule AD Bailey KR Turner ST. What is the goal with endogenous filtration markers–estimation of GFR or prediction of kidney outcomes? *Am J Kidney Dis.* (2011) 58:865–7. 10.1053/j.ajkd.2011.10.001 22099567

[8] Inker LA Titan S. Measurement and estimation of GFR for use in clinical practice: core curriculum 2021. *Am J Kidney Dis.* (2021) 78:736–49. 10.1053/j.ajkd.2021.04.016 34518032

[9] Kashani KB Frazee EN Kukrálová L Sarvottam K Herasevich V Young PM Evaluating muscle mass by using markers of kidney function: development of the sarcopenia index. *Crit Care Med.* (2017) 45:e23–9. 10.1097/ccm.0000000000002013 27611976

[10] Tabara Y Kohara K Okada Y Ohyagi Y Igase M. Creatinine-to-cystatin C ratio as a marker of skeletal muscle mass in older adults: J-SHIPP study. *Clin Nutr.* (2020) 39:1857–62. 10.1016/j.clnu.2019.07.027 31431305

[11] Jung CY Joo YS Kim HW Han SH Yoo TH Kang SW Creatinine-cystatin C ratio and mortality in patients receiving intensive care and continuous kidney replacement therapy: a retrospective cohort study. *Am J Kidney Dis.* (2021) 77:509.e–16.e. 10.1053/j.ajkd.2020.08.014 33098923

[12] Lin YL Chen SY Lai YH Wang CH Kuo CH Liou HH Serum creatinine to cystatin C ratio predicts skeletal muscle mass and strength in patients with non-dialysis chronic kidney disease. *Clin Nutr.* (2020) 39:2435–41. 10.1016/j.clnu.2019.10.027 31732290

[13] Oh KH Park SK Park HC Chin HJ Chae DW Choi KH KNOW-CKD (KoreaN cohort study for outcome in patients with chronic kidney disease): design and methods. *BMC Nephrol.* (2014) 15:80. 10.1186/1471-2369-15-80 24884708 PMC4050398

[14] Levey AS Stevens LA Schmid CH Zhang YL Castro AF III Feldman HI A new equation to estimate glomerular filtration rate. *Ann Intern Med.* (2009) 150:604–12. 10.7326/0003-4819-150-9-200905050-00006 19414839 PMC2763564

[15] Kalantari K Bolton WK. A good reason to measure 24-hour urine creatinine excretion, but not to assess kidney function. *Clin J Am Soc Nephrol.* (2013) 8:1847–9. 10.2215/cjn.09770913 24158794 PMC3817914

[16] Patel SS Molnar MZ Tayek JA Ix JH Noori N Benner D Serum creatinine as a marker of muscle mass in chronic kidney disease: results of a cross-sectional study and review of literature. *J Cachexia Sarcopenia Muscle.* (2013) 4:19–29. 10.1007/s13539-012-0079-1 22777757 PMC3581614

[17] Delanaye P Cavalier E Pottel H. Serum creatinine: not so simple! *Nephron.* (2017) 136:302–8. 10.1159/000469669 28441651

[18] Stevens LA Schmid CH Greene T Li L Beck GJ Joffe MM Factors other than glomerular filtration rate affect serum cystatin C levels. *Kidney Int.* (2009) 75:652–60. 10.1038/ki.2008.638 19119287 PMC4557800

[19] Zi M Xu Y. Involvement of cystatin C in immunity and apoptosis. *Immunol Lett.* (2018) 196:80–90. 10.1016/j.imlet.2018.01.006 29355583 PMC7112947

[20] Osaka T Hamaguchi M Hashimoto Y Ushigome E Tanaka M Yamazaki M Decreased the creatinine to cystatin C ratio is a surrogate marker of sarcopenia in patients with type 2 diabetes. *Diabetes Res Clin Pract.* (2018) 139:52–8. 10.1016/j.diabres.2018.02.025 29496508

[21] Hirai K Tanaka A Homma T Goto Y Akimoto K Uno T Serum creatinine/cystatin C ratio as a surrogate marker for sarcopenia in patients with chronic obstructive pulmonary disease. *Clin Nutr.* (2021) 40:1274–80. 10.1016/j.clnu.2020.08.010 32863062

[22] Ulmann G Kaï J Durand JP Neveux N Jouinot A De Bandt JP Creatinine-to-cystatin C ratio and bioelectrical impedance analysis for the assessement of low lean body mass in cancer patients: comparison to L3-computed tomography scan. *Nutrition.* (2021) 81:110895. 10.1016/j.nut.2020.110895 32739656

[23] Tabara Y Okada Y Ochi M Ohyagi Y Igase M. Association of creatinine-to-cystatin C ratio with myosteatosis and physical performance in older adults: the Japan shimanami health promoting program. *J Am Med Dir Assoc.* (2021) 22:2366.e–72.e. 10.1016/j.jamda.2021.03.021 33915077

[24] Potok OA Ix JH Shlipak MG Katz R Hawfield AT Rocco MV The difference between cystatin C- and creatinine-based estimated GFR and associations with frailty and adverse outcomes: a cohort analysis of the systolic blood pressure intervention trial (SPRINT). *Am J Kidney Dis.* (2020) 76:765–74. 10.1053/j.ajkd.2020.05.017 32682697 PMC8896529

[25] Kim H Park JT Lee J Jung JY Lee KB Kim YH The difference between cystatin C- and creatinine-based eGFR is associated with adverse cardiovascular outcome in patients with chronic kidney disease. *Atherosclerosis.* (2021) 335:53–61. 10.1016/j.atherosclerosis.2021.08.036 34571286

[26] Orlandi PF Huang J Fukagawa M Hoy W Jha V Oh KH A collaborative, individual-level analysis compared longitudinal outcomes across the international network of chronic kidney disease (iNETCKD) cohorts. *Kidney Int.* (2019) 96:1217–33. 10.1016/j.kint.2019.07.024 31570197

[27] Oh KH Kang M Kang E Ryu H Han SH Yoo TH The KNOW-CKD study: what we have learned about chronic kidney diseases. *Kidney Res Clin Pract.* (2020) 39:121–35. 10.23876/j.krcp.20.042 32550711 PMC7321679

[28] Rizk JG Streja E Wenziger C Shlipak MG Norris KC Crowley ST Serum. creatinine-to-cystatin-C ratio as a potential muscle mass surrogate and racial differences in mortality. *J Ren Nutr.* (2021) 21:S1051–2276. 10.1053/j.jrn.2021.11.005 34923112

