# Supplementary material for: Serum creatinine to cystatin C ratio and clinical outcomes in adults with non-dialysis chronic kidney disease
Source: Front Nutr. 2022 Sep 26;9:996674. doi: 10.3389/fnut.2022.996674 (PMC9550211; doi:10.3389/fnut.2022.996674)
Supplement: Supplementary file 1 [file Data_Sheet_1.pdf]

Supplementary Material

SUPPLEMENTARY TABLES AND FIGURES

Supplementary Table S1 | Linear Regression Models of the Creatinine/Cystatin C Ratio in Adults with Chronic Kidney Disease

	Unadjusted Association		Model Adjusted for Age, Sex, eGFR, and ACR		Fully Adjusted Model	
**Covariates**	**β (95% CI)**	***P***	**β (95% CI)**	***P***	**β (95% CI)**	***P***
Age, years	-0.029 (-0.369 to -0.022)	<0.001	-0.059 (-0.065 to -0.052)	<0.001	-0.051 (-0.058 to -0.044)	<0.001
Sex, female	-1.872 (-2.041 to -1.702)	<0.001	-1.834 (-1.988 to -1.680)	<0.001	-1.557 (-1.751 to -1.364)	<0.001
eGFR, mL/min/1.73m^2	-0.014 (-0.017 to -0.012)	<0.001	-0.026 (-0.029 to -0.023)	<0.001	-0.027 (-0.030 to -0.044)	<0.001
Ln UACR, mg/g	-0.055 (-0.103 to -0.007)	0.03	-0.167 (-0.209 to -0.125)	<0.001	-1.105 (-0.153 to -0.568)	<0.001
24-h Urine creatinine, 100mg/day	0.187 (0.166 to 0.208)	<0.001	-		0.096 (0.072 to 0.120)	<0.001
Ln C-reactive protein, mg/L	-0.070 (-0.137 to -0.004)	0.04	-		-0.127 (-0.183 to -0.072)	<0.001
Albumin, g/dL	0.497 (0.281 to 0.714)	<0.001			0.356 (0.148 to 0.563)	<0.001
Diabetes	-0.112 (-0.307 to 0.082)	0.2	-		0.042 (-0.130 to 0.216)	0.6
Cardiovascular disease	-0.143 (-0.367 to 0.110)	0.2	-		-0.275 (-0.487 to -0.064)	<0.001
Current smoker	0.210 (-0.049 to 0.468)	0.1	-		-0.597 (-0.815 to -0.380)	<0.001
Body mass index, kg/m^2	0.018 (-0.009 to 0.046)	0.2	-		-0.012 (-0.036 to 0.011)	0.3
R^2	not available		0.339		0.391	

CI: confidence interval; eGFR: estimated glomerular filtration rate; UACR: urine albumin-creatinine ratio; Ln: natural log.

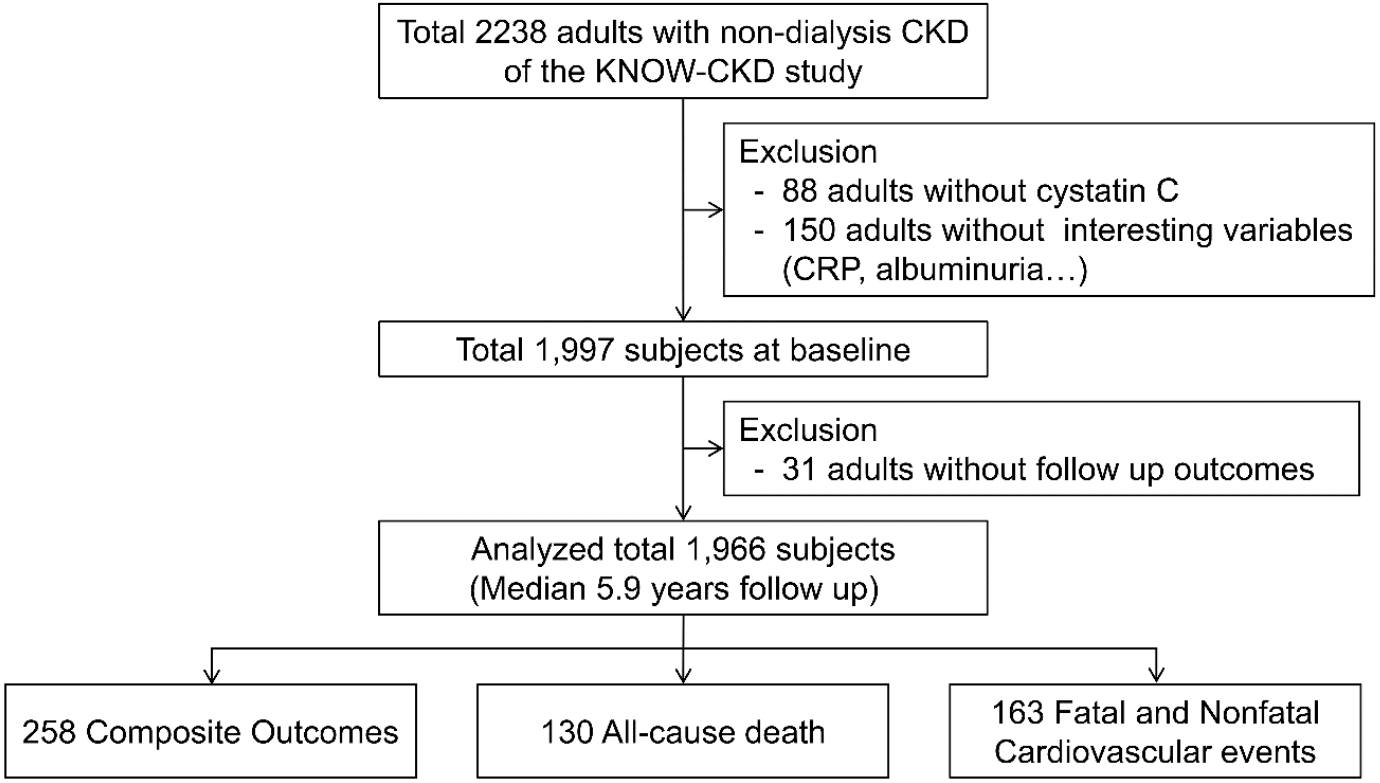

Supplementary Figure S1 | Flowchart of the enrolled study population.
CKD: chronic kidney disease; CRP: C-reactive protein; KNOW-CKD: KoreaN cohort study Outcomes in patients With CKD.

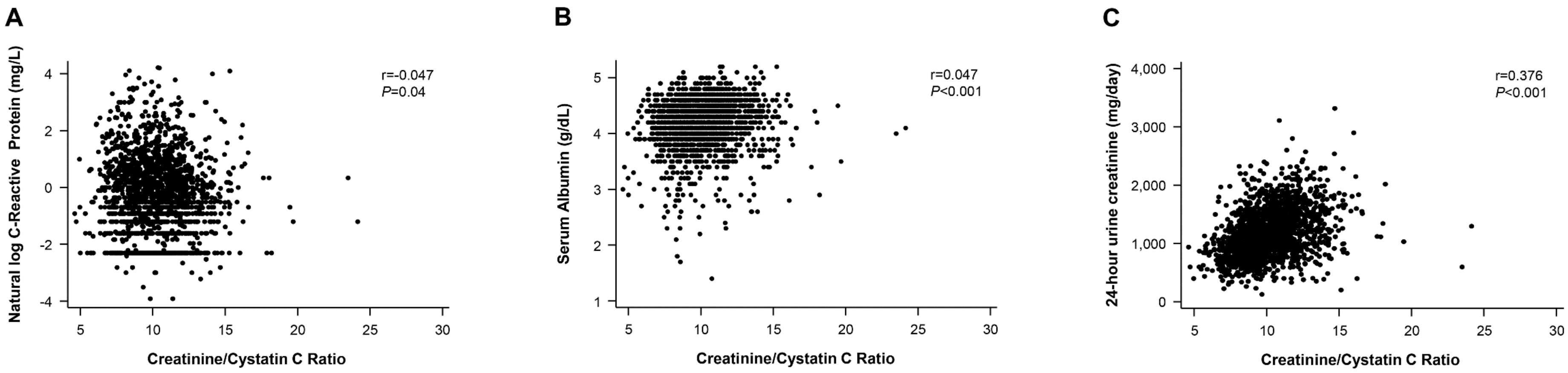

Supplementary Figure S2 | A scatterplot and correlations between the Cr/CysC ratio and (A) the natural logs of C-reactive protein, (B) serum albumin, and (C) 24-hour urine creatinine.

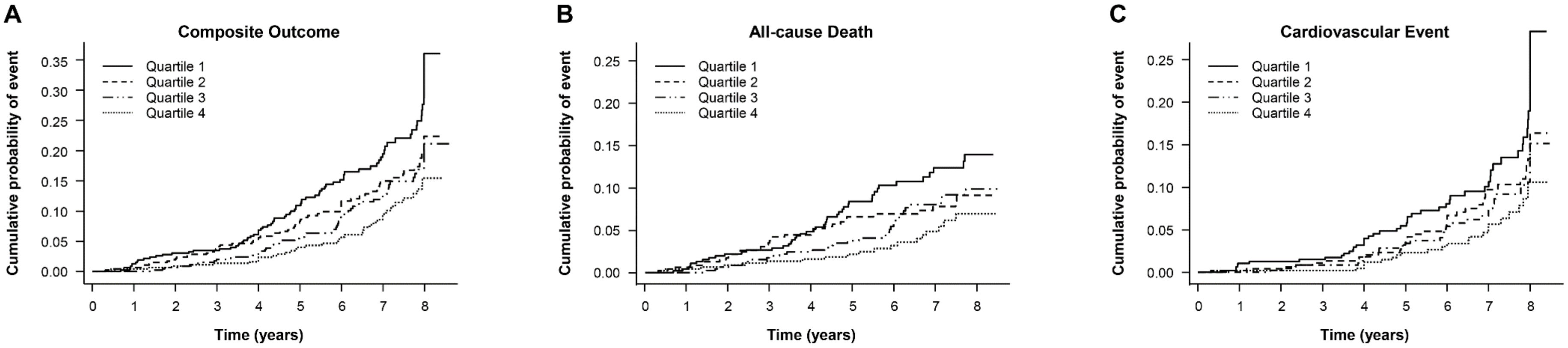

Number at risk

Quartile 1	492	460	435	414	374	279	214	154	68
Quartile 2	491	470	450	421	402	318	254	193	86
Quartile 3	492	479	459	444	415	340	265	208	98
Quartile 4	491	459	436	418	398	329	269	195	85

Number at risk

Quartile 1	492	460	435	414	374	279	214	154	68
Quartile 2	491	470	450	421	402	318	254	193	86
Quartile 3	492	479	459	444	415	340	265	208	98
Quartile 4	491	459	436	418	398	329	269	195	85

Number at risk

Quartile 1	492	460	435	414	374	279	214	154	68
Quartile 2	491	470	450	421	402	318	254	193	86
Quartile 3	492	479	459	444	415	340	265	208	98
Quartile 4	491	459	436	418	398	329	269	195	85

Supplementary Figure 3 | Cumulative composite outcomes, all-cause death, and cardiovascular events probability according to creatinine/cystatin C ratio quartiles. Kaplan-Meier curve of (A) composite outcomes, (B) all-cause death, and (C) cardiovascular events stratified to creatinine/cystatin C ratio quartiles. Log-rank *P*<0.01 for quartile 1 versus other groups.
